# 

**Published:** 1879-05

**Authors:** 


					﻿MAY, 1879.
TWENTY-SIXTH YEAR—No. 305.
ll.VLL^
Journal of Health.
PUBLISHED AT 141 EIGHTH STREET,
(Near Broadway),	JSTZETW" YORK.
E. H. GIBBS, A.M., M.D., Editor.
AGENTS FOR THE TRADE:
AMERICAN NEWS COMPANY AND NEW YORK NEWS COMPANY
Terms, $1.50 a Year, or $1.00 for Eight Months. Postage paid.
SINGLE NUMBER, 15 CENTS.
Couch & Johnston, Printers, 11 Frankfort Street, New York.
				

